# Miltefosine Resistant Field Isolate From Indian Kala-Azar Patient Shows Similar Phenotype in Experimental Infection

**DOI:** 10.1038/s41598-017-09720-1

**Published:** 2017-09-04

**Authors:** Supriya Khanra, Nibedeeta R. Sarraf, Anjan K. Das, Syamal Roy, Madhumita Manna

**Affiliations:** 1 Department of Zoology, Barasat Govt. College, 10, K.N.C Road, Kolkata, 700124 India; 2 Department of Pathology, Calcutta National Medical College, 32, Gorachand Road, Kolkata, 700014 India; 3 Department of Infectious Diseases & Immunology, Indian Institute of Chemical Biology, 4, Raja S.C. Mullick Road, Kolkata, 700032 India; 4 Present Address: Crystallography and Molecular Biology Division, Saha Institute of Nuclear Physics, 1/AF Bidhannagar, Kolkata, 700064 India; 5 Present Address: Cooch Behar Panchanan Barma University, Vivekananda Road, Cooch Behar, West Bengal, 736101 India; 6 Present Address: Bidhannagar College, EB 2, Salt Lake, Sector I, Kolkata, 700064 India

## Abstract

Emergence of resistance to drugs used to treat the Indian Kala-azar patients makes control strategy shattered. In this bleak situation, Miltefosine (MIL) was introduced to treat mainly antimonial unresponsive cases. Within years, resistance to MIL has been reported. While checking the MIL sensitivity of the recent KA clinical isolates (n = 26), we came across one isolate which showed four times more EC_50_ for MIL than that of MIL-Sensitive (MIL-S) isolates and considered as putative MIL-Resistant (MIL-R). The expressions of LdMT and LdRos3 genes of this isolate were found down regulated. Th1/Th2 cytokines, ROS and NO, FACS dot plots and mitochondrial trans membrane potential measurement were performed. *In vivo* hamster model with this MIL-R isolate showed much lesser reduction in liver weight (17.5%) compared to average reduction in liver weight (40.2%) of the animals infected with MIL-S isolates. The splenic and hepatic stamps smears of MIL-R infected hamsters revealed the retention of parasite load of about 51.45%. The splenocytes of these animals failed to proliferate anti leishmanial T-cells and lack of cell mediated immunity hampered recovery. Thus, these phenotypic expressions of experimental model may be considered similar to that of the MIL unresponsive patients. This is first such kind of report.

## Introduction

The disease, Visceral Leishmaniasis (VL) or Kala-azar (KA) is endemic in the Indian subcontinent and broadening its base on the Gangetic plains of Bangladesh, India and Nepal. KA is the most fetal disease if left untreated^1–5^. Occurrence of immune suppression in the host (eg, human immunodeficiency virus [HIV] co infection) and emergence of resistance to first line antimonial drugs^6, 7^ are two serious health problems associated with the disease. Earlier reports suggested that resistance of *Leishmania* parasites to antimonials is related to Sodium Stibo Gluconate (SSG) treatment failure in the Indian state, Bihar^8, 9^. Second line treatment with Amphotericin B (AmB) was highly efficacious^10^ but unresponsiveness towards AmB have started been reported^11^. Other substitute drugs such as Pentamidine, Paromomycin remain largely inadequate due to high cost, high toxicity or side effects^2, 12^. The existing oral drug Miltefosine (MIL) (hexadecylphosphocholine), a lysophospholipid analog, was mainly developed as an anticancer drug but it is effective *in vitro* against a variety of species of *Leishmania* ^13, 14^ and other protozoan parasites including *Trypanosoma cruzi*, *T.brucei* ^15^, *Entamoeba histolytica* ^16^ and *Acanthamoeba sp* ^17^. MIL, had been approved in India, in 2002, for the treatment of Indian VL patients^18^, including cases unresponsive to antimonial and has achieved more than 97% cure rate^19^. Within few years, unresponsiveness to MIL in VL patients^20, 21^ and failure of MIL in the treatment of KA in Nepal have been reported^22^. The above observations revealed the crude reality that any drug, how efficacious it may be, after some years of its use, resistance would appear. Thus, the parasite resistance to the commonly used drugs must be monitored carefully to combat this deadly disease. For this, each and every field isolate of the patients should be characterized extensively and it is not done in many places including India.

For MIL transport in *Leishmania* parasites, a two-subunit amino phospholipid translocase, *Leishmania donovani* miltefosine transporter (LdMT) and its specific beta subunit LdRos3, internalizes the drug^23^. Phospholipid vesicles (liposomes) employed as carrier systems for MIL, reduces its toxic side effects^23^ and there have connection between the expression levels of both proteins and the parasite sensitivity towards the drug^24, 25^.

Understanding the mechanism of resistance, factors related to it and control strategy to develop thereafter against the resistant parasites, it is prerequisite to study MIL-R isolates *in vitro* and/or *in vivo*. In recent report, two Leishmania isolates were identified as MIL-Resistant (MIL-R) by the *in vitro* and genome study and these isolates were collected from confirmed Indian KA patients^26^. Few years back, researchers in the field generated a MIL-R *Leishmania* parasite by step-wise increment of drug pressure *in vitro* and reported that in case of MIL-Sensitive (MIL- S) *Leishmania* parasite, the effect of MIL was mediated through Apoptosis- like death but not in MIL-R *Leishmania* parasite^27^. Till date, there is no report of animal models for *in vivo* characterization of the MIL-R isolates. The phenotypic expression observed in the animal model may be similar to that of the unresponsive MIL patients and would be instrumental in developing the control strategy.

In the present study, we have characterized all the clinical isolates of Indian KA at species level because of the fact that though *Leishmania donovani* historically known as the causative agent for Indian KA or VL^3, 4^, other species (*L. tropica)* is found to be associated with the disease^5, 28^. Thus, before going for any typological work with any clinical isolate, it became mandate to ascertain its identification at species level with the help of species specific markers [e.g., rRNA gene-internal transcribed spacers (ITS), heat shock protein of 70 kDa (hsp70), Major surface protease msp (gp63) gene and genes encoding cysteine proteinase B etc]^29–32^. As a part of our epidemiological search for Indian KA, we rigorously characterized all the recently collected clinical isolates through Randomly amplified polymorphic DNA (RAPD) analysis and performed Restriction Fragment Length Polymorphism (RFLP) analysis with the help of several species specific markers (ITS1 and hsp70) to ascertain their species identity^4, 5^. All the isolates used here (n = 26) were collected from confirmed Indian KA and typed as *Leishmania donovani* ^4, 5^. Then we have checked the drug sensitivity of all isolates (n = 26) for MIL and found one as putative MIL-R field isolate of KA (*L. donovani*) with four times more EC_50_ for MIL than that of MIL-Sensitive (MIL-S) isolates. We have opted for *in vivo* evaluation of this field isolate (study code T9) in hamster model. Infection with this MIL-R isolate in hamsters showed 17.5% reduction in liver weight compared to average reduction in liver weight by 40.2% of the animals infected with MIL-S isolates. MIL-R infected animals revealed the retention of parasite load in spleen and liver by about 51.45% respectively. The splenocytes of these animals failed to proliferate anti leishmanial T-cells and this T-cell anergy hampered recovery mimicking the scenario with MIL unresponsive patients. Thus, we are reporting for the first time, the phenotypic expressions of confirmed MIL-R *L.donovani* isolate in hamster model.

## Results

### Identification of the clinical isolates at species level by Restriction Fragment Length Polymorphism (RFLP) method

The Internal Transcribed Spacer 1 (ITS1) RFLP^33^ and the Heat Shock Protein 70 (hsp70) RFLP^32^ are well-known molecular markers for the characterization of *Leishmania* parasites at species level. The ITS1 region and hsp70 region of all the samples were amplified separately and subjected to ITS1 RFLP and hsp70 RFLP analysis individually. Species-specific RFLP patterns were obtained for *L.donovani* WHO strain (DD8) and *L. tropica* WHO strain (K27) respectively. A portion of the isolates of the present study, had been characterized earlier by RAPD (n = 9)^4^ and RFLP (n = 25)^5, 34^ methods. We noticed that the additional clinical isolate (n = 1) of the present study have shown similar ITS1 RFLP (Supplementary Fig. S1) and hsp70 RFLP profile (Supplementary Fig. S2) as that of *L. donovani* standard strain DD8. Thus, the clinical isolates along with the putative MIL-R (study code T9) included in the present study were identified as *L. donovani*.

### *In vitro* MIL susceptibility assay

MIL susceptibility was determined at intracellular amastigote stage for the field isolates of KA. In the present study, we have used RAW 264.7 cells as host cell and percentage of infected MØs ranged from 80 to 89 and the number of amastigotes/100 MØs ranged from 90 to 98. When the intracellular amastigotes of the KA isolates were subjected to test the susceptibility towards MIL, the EC_50_ values ranged from 1.74 ± 0.10 to 5.35 ± 0.83 μM with a mean EC_50_ of 3.32 ± 0.07 μM for MIL-sensitive (MIL-S) isolates while only one field isolate, T9 showed EC_50_ of 13.47 ± 0.87 μM (Table 1) which was approximately 4 times more EC_50_ value compared to the mean EC_50_ value of the MIL-S isolates. On the other hand, the AG83 (MHOM/IN/1983/AG83) isolate was used as a reference strain as it has been thoroughly worked out by workers as sensitive towards both of the drugs: MIL^35^ and Sodium Stibo Gluconate (SSG)^36^. Our single MIL-resistant (MIL-R) isolate from the field, showed approximately 3 times higher EC_50_ value compared to the EC_50_ value of the *L. donovani* MIL-S isolate (AG83).

**Table 1:** Susceptibility of intracellular amastigotes of *Leishmania* isolates to Miltefosine (MIL), represented by EC_50_ values.

Serial No.	Isolates	MIL EC_50_ ± SD (µM)
1	AG83	4.39 ± 0.43
2	T2	4.69 ± 0.28
3	T3	2.39 ± 0.025
4	T4	3.73 ± 1.34
5	T7	2.47 ± 0.13
6	M1	3.88 ± 0.33
7	PG2	3.99 ± 0.59
8	PG3	2.28 ± 0.11
9	PG4	3.78 ± 0.47
10	RAJ-04	2.44 ± 0.58
11	RAJ-05	2.70 ± 0.58
12	RAJ-07	3.09 ± 0.27
13	P1	4.24 ± 0.15
14	BI	3.91 ± 0.89
15	BHU581	2.14 ± 0.33
16	BHU569	2.09 ± 0.48
17	BHU568	2.08 ± 0.41
18	BHU572	1.89 ± 0.26
19	BHU573	3.19 ± 0.48
20	BHU574	1.74 ± 0.10
21	BHU575	2.75 ± 0.41
22	BHU592	2.80 ± 0.24
23	BHU860	4.66 ± 0.82
24	BHU965	5.35 ± 0.83
25	T8	4.29 ± 0.59
26	P2	3.66 ± 0.47
27	T9	13.47 ± 0.87

Serial number in bold and regular denoted unresponsive (MIL-R) and sensitive (MIL-S) to MIL, respectively. All the isolates were MIL-S except serial no. 27, denoting MIL-R isolate (T9). Results were given as mean ± SD.

This observation corroborated the previous report of two MIL-R isolates^26^, where the researchers suggested that the IC_50_ value of MIL-R field isolates approximately 2 times more higher than the IC_50_ value of the *L. donovani* MIL-S standard strain (DD8)^26^ although it is also reported that, the *in vitro* activity regarding the effectiveness of any anti-leishmanial drugs against Leishmanial parasites may be dependent on the host cell^37^. Our putative MIL-R isolate (T9) is SSG-Sensitive (SSG-S) with EC_50_ value 4.69 ± 0.30 μg/ml and the result was also expressed in terms of Activity Index (AI) using AG83 as reference isolate of KA. Isolates with an AI ≥ 3.0 are considered as SSG-Resistant (SSG-R) and an AI < 3 are considered as SSG-Sensitive (SSG-S)^34, 38^. The AI of the MIL-R isolate is 2.51. Therefore it was identified as SSG-S.

### Expression of cytokines level

**Figure 1:**
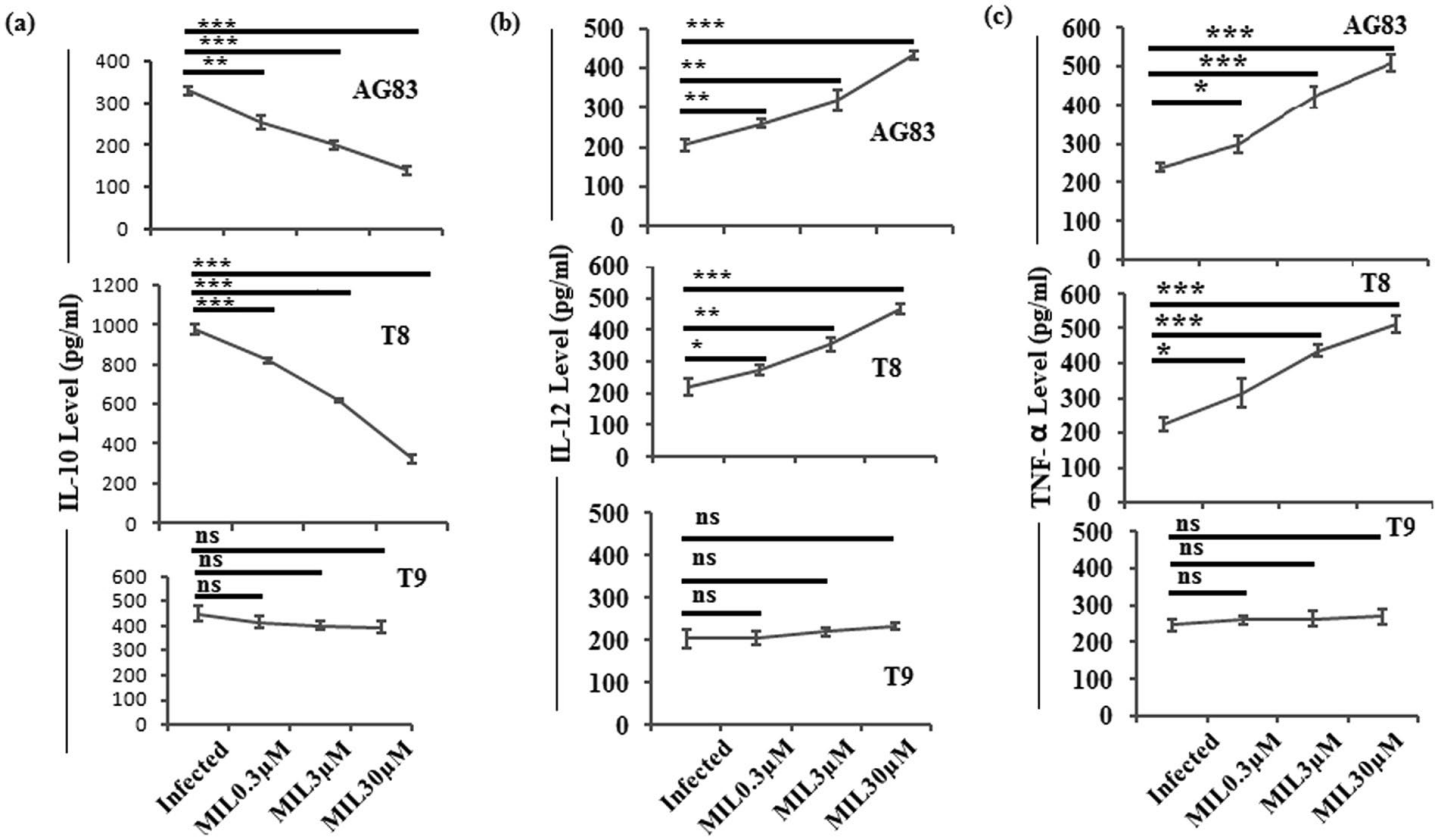
Analysis of cytokines level releases from macrophages (MØs) infected with the clinical isolates (AG83, T8, T9) following different concentrations of MIL treatment. The culture supernatants were harvested to determine (**a**) IL-10, (**b**) IL-12 and (**c**) TNF-α content by ELISA. Data represent mean ± SD of three independent experiments. *P < 0.05,**P < 0.001, ***P < 0.0001(Student’s t-test), representing significant differences between clinical isolate infected macrophages and MIL treated infected macrophages in (**a**) IL-10, (**b**) IL-12 and (**c**) TNF-α cytokine production respectively. NS: Non significance.

In case of MØs infected with MIL-S clinical isolates (Fig. 1a panels AG83, T8), the expression level of IL-10 were gradually decreased with increasing concentrations of MIL. On the other hand, there was no significant decrease in the expression level of IL-10 release from MØs infected with putative MIL-R isolate (T9) and then treated with MIL (Fig. 1a).

On the other hand, the expression levels of IL-12 (Fig. 1b panels AG83, T8) and TNF-α (Fig. 1c panels AG83, T8) releases from MØs infected with MIL-S KA isolates following drug treatment (MIL), were gradually increased with increasing concentrations of MIL. On contrary, there is no significant change in the expression levels of IL-12 (Fig. 1b, panel T9) and TNF-α (Fig. 1c, panel T9) releases from MØs infected with putative MIL-R isolate and then treated with MIL with respect of its infected control group.

### Measurement of nitric oxide (NO) and reactive oxygen species (ROS)

The *in vitro* MIL sensitivity assay and cytokines data showed the response patterns of the studied clinical isolates towards MIL. We carried out experiments to understand the status of NO and ROS in infected and drug treated macrophages because they are the essential leishmanicidal molecules as stated earlier^39^.

**Figure 2:**
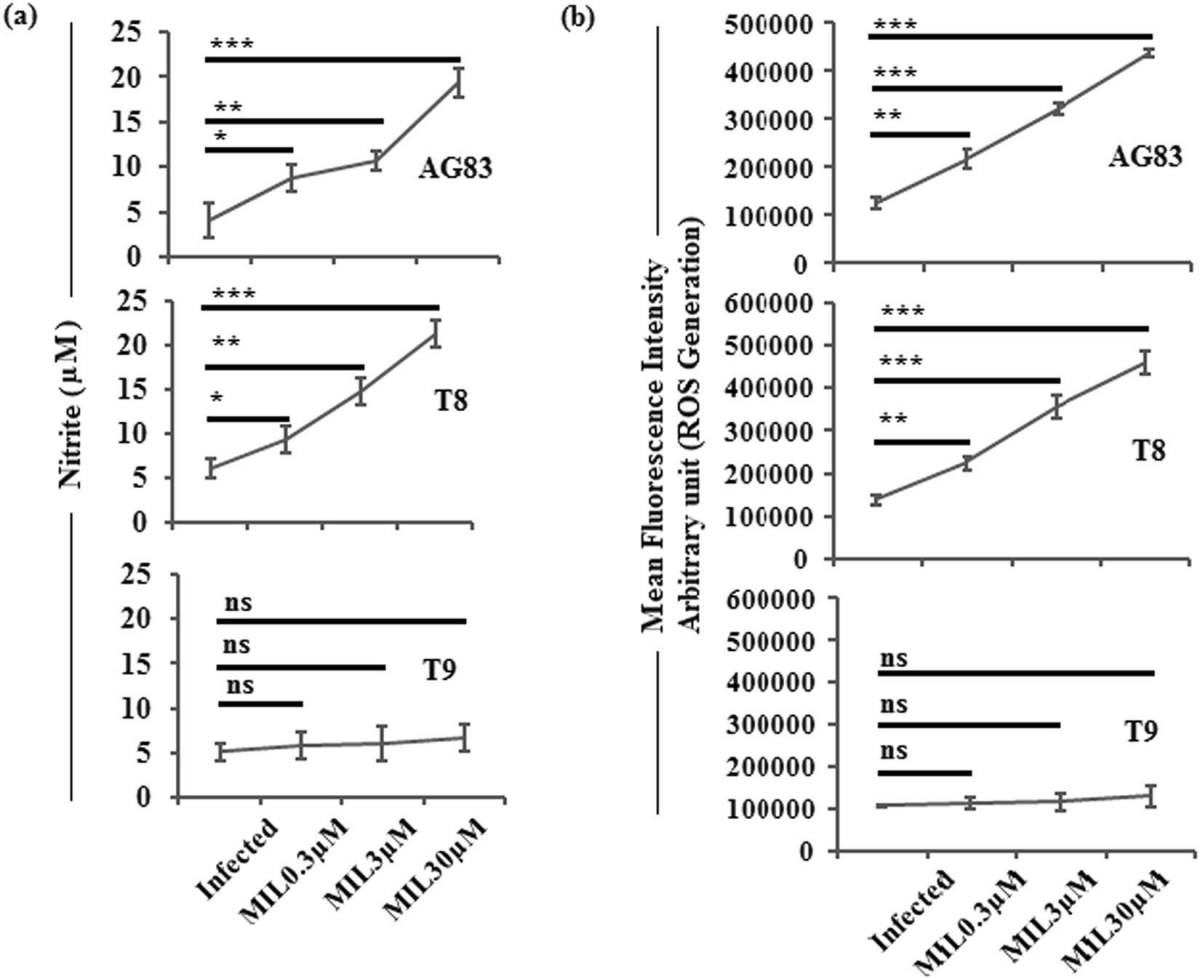
Generation of Nitrite production and ROS in the studied isolates (AG83, T8, T9) infected and MIL treated macrophages. Culture supernatant was used to evaluate (**a**) NO generation by Griess method and (**b**) ROS generation was measured by H_2_DCFDA probe. Data represents mean ± SD of three independent experiments; *P < 0.05,**P < 0.001, ***P < 0.0001(Student’s t-test), representing significant differences between studied isolate infected macrophages and MIL-treated infected macrophages in (**a**) NO and (**b**) ROS production respectively. NS: Non significance.

In case of MIL-S clinical isolates (AG83 & T8) infected MØs, the generation levels of NO (Fig. 2a panels AG83, T8) and ROS (Fig. 2b panels AG83, T8) were gradually enhanced with increasing concentrations of MIL. There was no significant increase in the NO (Fig. 2a, panel T9) and ROS (Fig. 2b, panel T9) releases from MØs infected with putative MIL-R isolate (T9) and treated with MIL with respect to infected control group.

### Detection of the mitochondrial trans membrane potential (ΔΨm) and viable *Leishmania* isolates following MIL treatment

**Figure 3:**
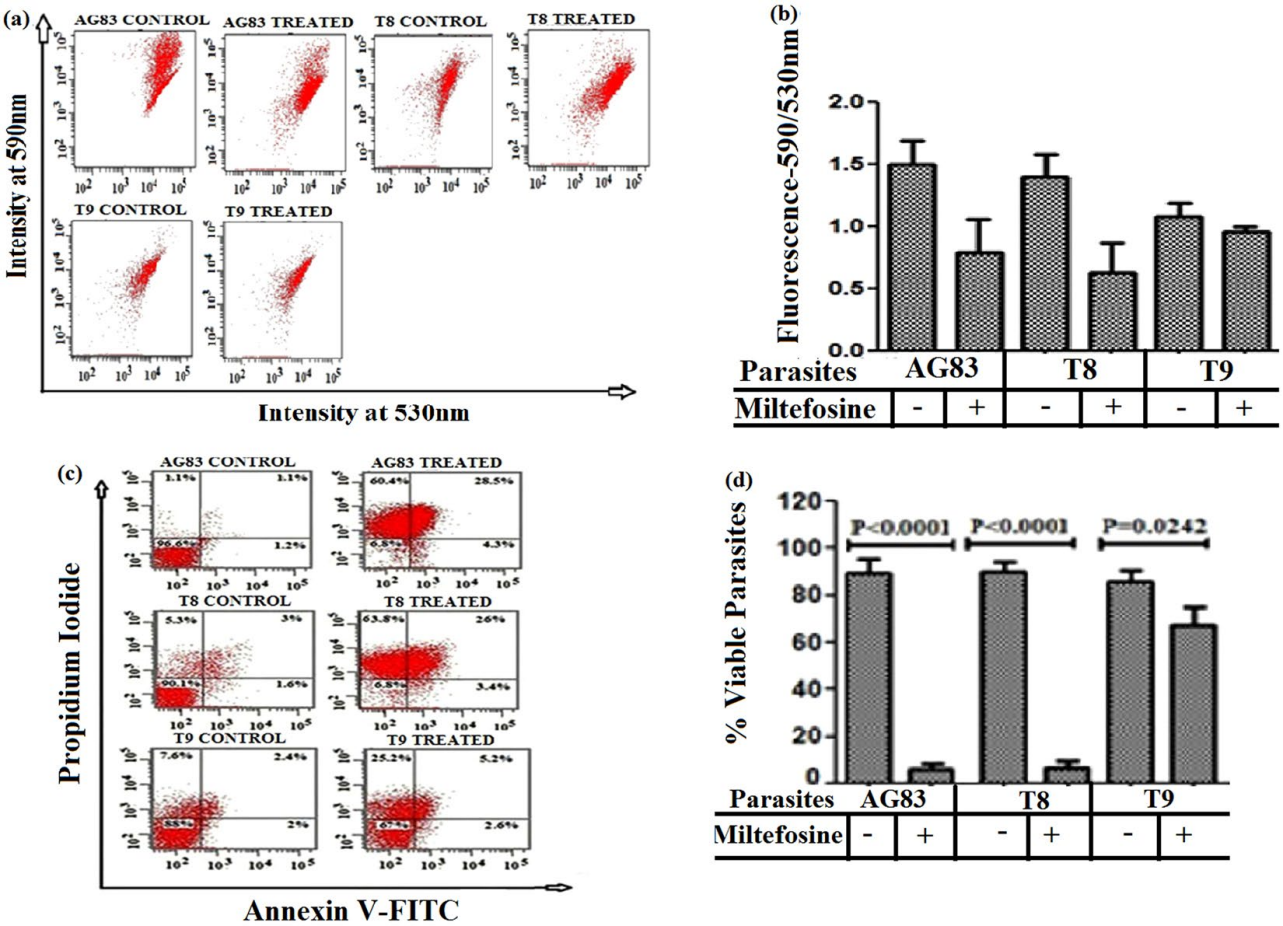
Assessment of mitochondrial transmembrane potential in different clinical isolates (AG83, T8, T9) and viable *Leishmania* isolates following MIL treatment. (**a**) Mitochondrial membrane potential (ΔΨm) in studied isolates following MIL treatment were measured using 5,5,6,6-Tetrachloro-1,1,3,3-tetraethylbenzimidazole carbocyanide iodide (JC-1) probe and (**b**) the bar graph showing the ratio of fluorescence (590 nm/530 nm) measured by fluorescence activated cell sorting analysis. (**c**) *A*nnexin V fluorescein isothiocyanate (FITC)- Propidium Iodide(PI) Assay in the studied isolates following MIL treatment were performed to assess viable *Leishmania* isolates and the lower left quadrant of each dot plot represents viable isolates. (**d**) The bar graph showing the viable cells as determined by fluorescence activated cell sorting analysis. Data are expressed as the mean ± SD of three independent experiments and levels of significance are indicated by P values.

JC-1 was used as a probe for the measurement of ΔΨm by flow cytometry. We observed that the MIL-S clinical isolates (AG83, T8) showed sensitivity to MIL as evident from the noteworthy decrease in ΔΨm after 72 h of MIL treatment (Fig. 3a,b panels AG83, T8). When the putative MIL-R isolate (T9) was exposed to similar MIL treatment, the red/green fluorescence ratio was not decreased significantly due to the less sensitivity towards MIL (Fig. 3a,b panel T9).

The flow cytometric analysis with FITC Annexin V/PI double staining result revealed that when MIL-S clinical isolates were exposed to MIL, around 7% viable population of cells were observed (Fig. 3c,d panels AG83, T8). On contrary, after MIL exposure to T9 (putative MIL-R) about 67% viable population of cells were observed (Fig. 3c,d panel T9).

### LdMT and LdRos3 expressions in different field isolates

**Figure 4:**
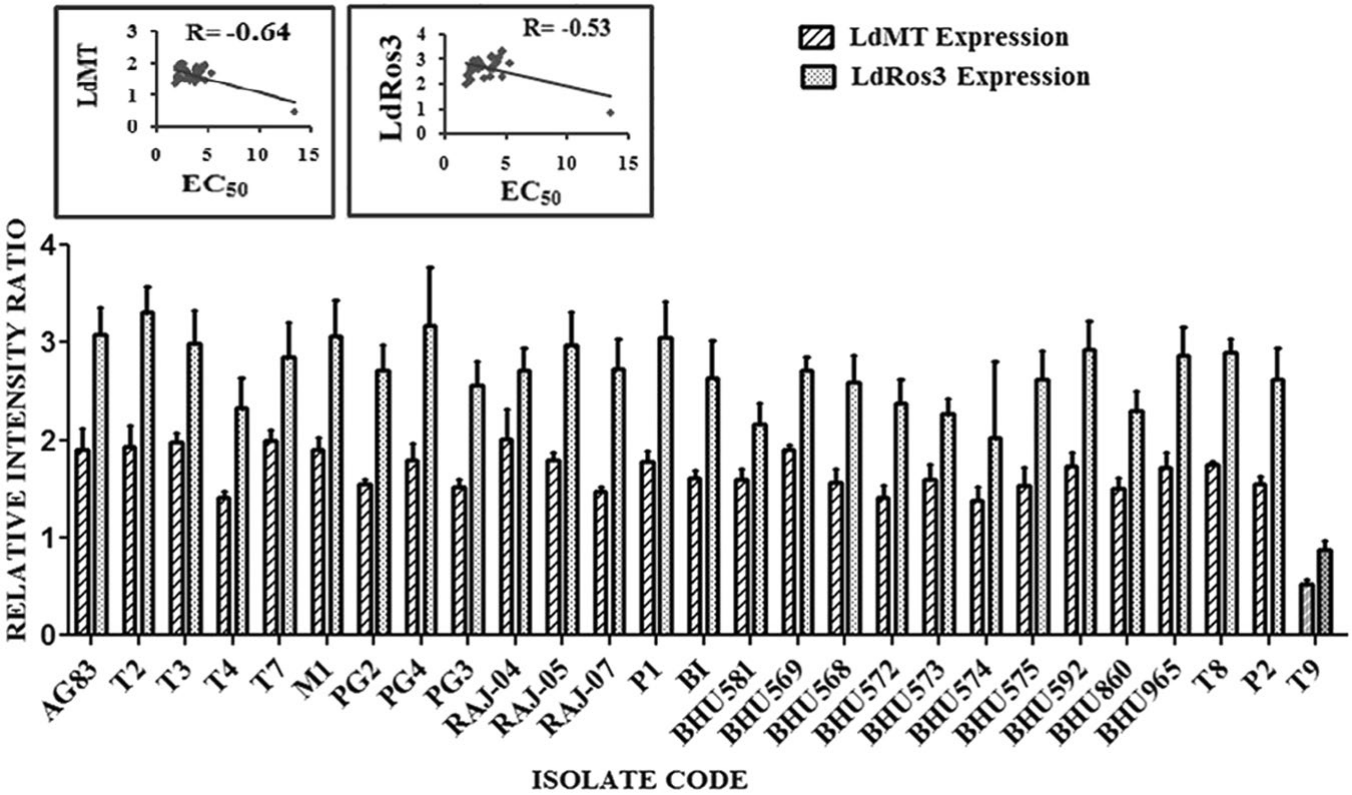
Graphical representation of the densitometric data. Expression level of LdMT was expressed as a ratio of LdMT mRNA level to GAPDH mRNA level and the expression level of LdRos3 was expressed as a ratio of LdRos3 mRNA level to GAPDH mRNA level. The levels of expression of LdMT and LdRos3 were correlated and LdRos3 expression was higher than LdMT expression. Data were expressed as the mean ± SD of three independent experiments. Each scatter plot in the inset represents the correlation between the EC_50_ of the isolates against MIL and the respective gene expression.

The expression level of the MIL transporter LdMT and its beta subunit LdRos3 were determined using semi quantitative Reverse Transcription (RT)-PCR analysis (Supplementary Fig. S3). LdMT expression was approximately 3.22 times higher in MIL-S isolates (r = −0.64) and the LdRos3 has also shown approximately 3.11 fold higher expression in MIL-S isolates (r = −0.53). The levels of expression of LdMT and LdRos3 were correlated (Fig. 4).

### Measurement of Multidrug resistant protein A (MRPA) expression level

The expression level of MRPA was determined by semi quantitative Reverse Transcription (RT)-PCR analysis (Supplementary Fig. S4). It has been revealed that the single MIL-R field isolate (T9) showed approximately 3.4 times higher level MRPA expression than that of the MIL-S *L. donovani* isolate (AG83) (Supplementary Fig. S4).

### Organomegally study in hamster model

**Figure 5:**
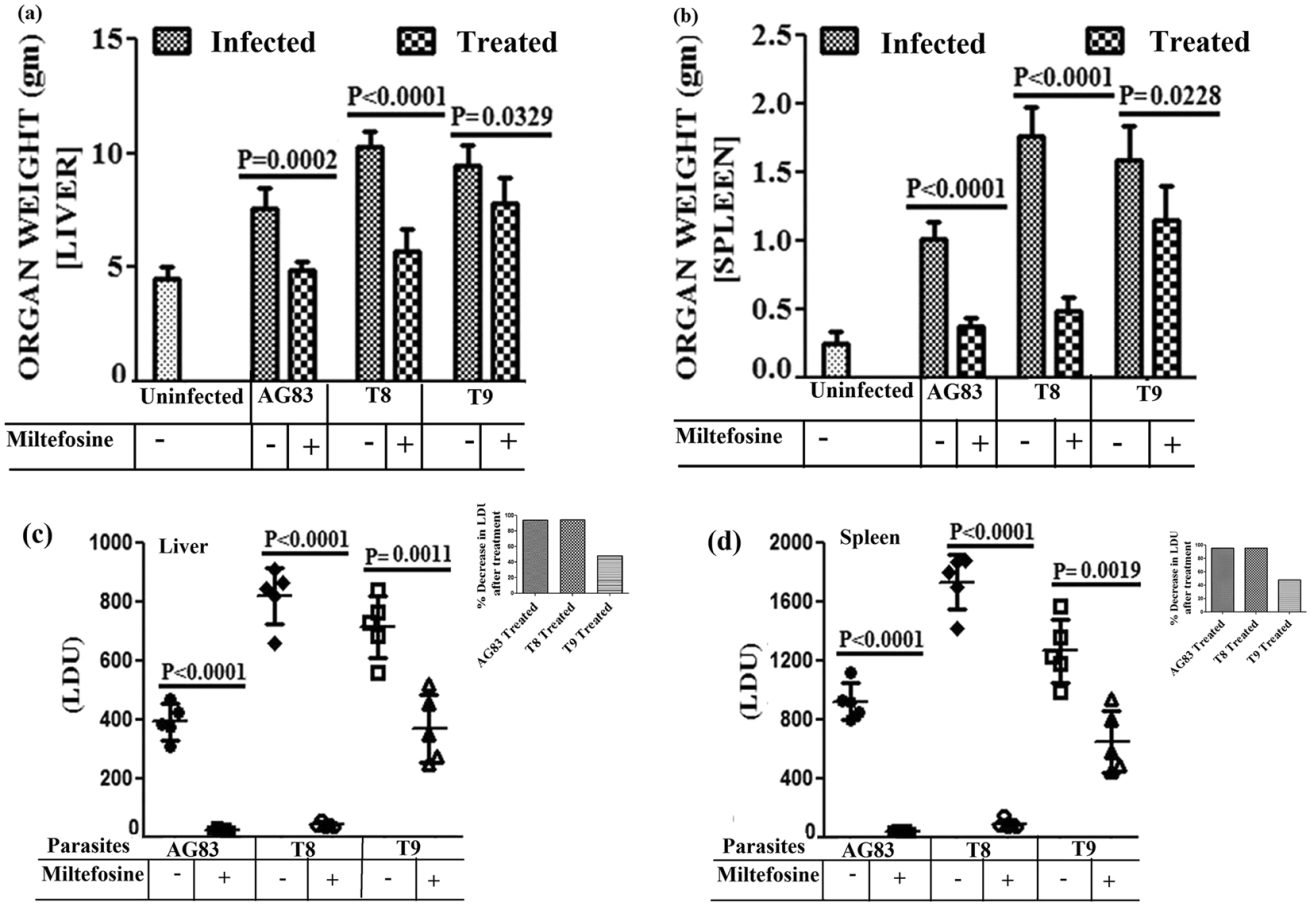
Study of Organomegaly and Estimation of parasite burden in the spleen and liver of the experimental groups of animals. Hamsters were infected with studied parasites and infection was allowed to establish for next 8 weeks. 8 weeks infected hamsters received MIL doses. After 45 days post treatment hamsters were sacrificed and (**a**) Liver weight and (**b**) Spleen weight of the hamsters of different experimental groups (AG83-INF, AG83-TRE; T8-INF, T8-TRE; T9-INF, T9-TRE) were determined. Data represents mean ± SD of 5 animals per group; unpaired two-tailed Student’s t-test was performed and levels of significance are indicated by P values. On the other hand, after 45 days post treatment hamsters were sacrificed and the (**c**) hepatic as well as the (**d**) splenic parasite load was determined by stamps-smear method. Total parasite load in each organ is expressed in LDU unit. 1 LDU = amastigote per nucleated cell x organ weight in milligram. Data represents mean ± SD of 5animals per group; unpaired two-tailed Student’s t-test was performed and levels of significance are indicated by P values.

Liver weight of the treated groups of hamsters (AG83-TRE, T8-TRE and T9-TRE) showed reduction in weight by 35.99%, 44.38% and 17.48% respectively in comparison to that of the infected groups (AG83-INF, T8-INF and T9-INF) (Fig. 5a panels AG83, T8, T9). The spleen weights of the treated groups were decreased by 63.2%, 72.3% and 27.75% respectively with respect to that of the infected groups of hamsters (Fig. 5b panels AG83, T8, T9).

### Parasite load of liver and spleen were expressed as Leishman-Donovan Unit (LDU)

In liver, the parasite load after MIL treatment in experimental animal groups (AG83-TRE & T8-TRE) showed about 3–7% retention compared to that of the infected groups (Fig. 5c, panels AG83, T8) while group T9-TRE animals (infected with putative MIL-R isolate and then treated with MIL) showed retention of 32–62% (average 51.54%) parasites in the liver (Fig. 5c, panel T9). The retention of parasite load after MIL treatment in experimental animal groups (AG83-TRE & T8-TRE) of animals was about 3–8% in spleen compared to that of the infected groups (Fig. 5d panels AG83, T8). T9-TRE animals (infected with putative MIL-R isolate and then treated with MIL) showed retention of 36–69% (average 51.35%) parasites in spleen (Fig. 5d, panel T9).

### T-cell proliferation assay

The splenocytes of treated MIL-S groups (AG83-TRE & T8-TRE) stimulated with SLA showed a significantly higher level of T-cell proliferation than that of the infected groups and the splenocytes of T9-TRE group animals failed to proliferate anti leishmanial T-cell against leishmanial antigen at significantly higher level than T9-INF group (Supplementary Fig. S5).

### Measurement of antileishmanial antibody responses

**Figure 6:**
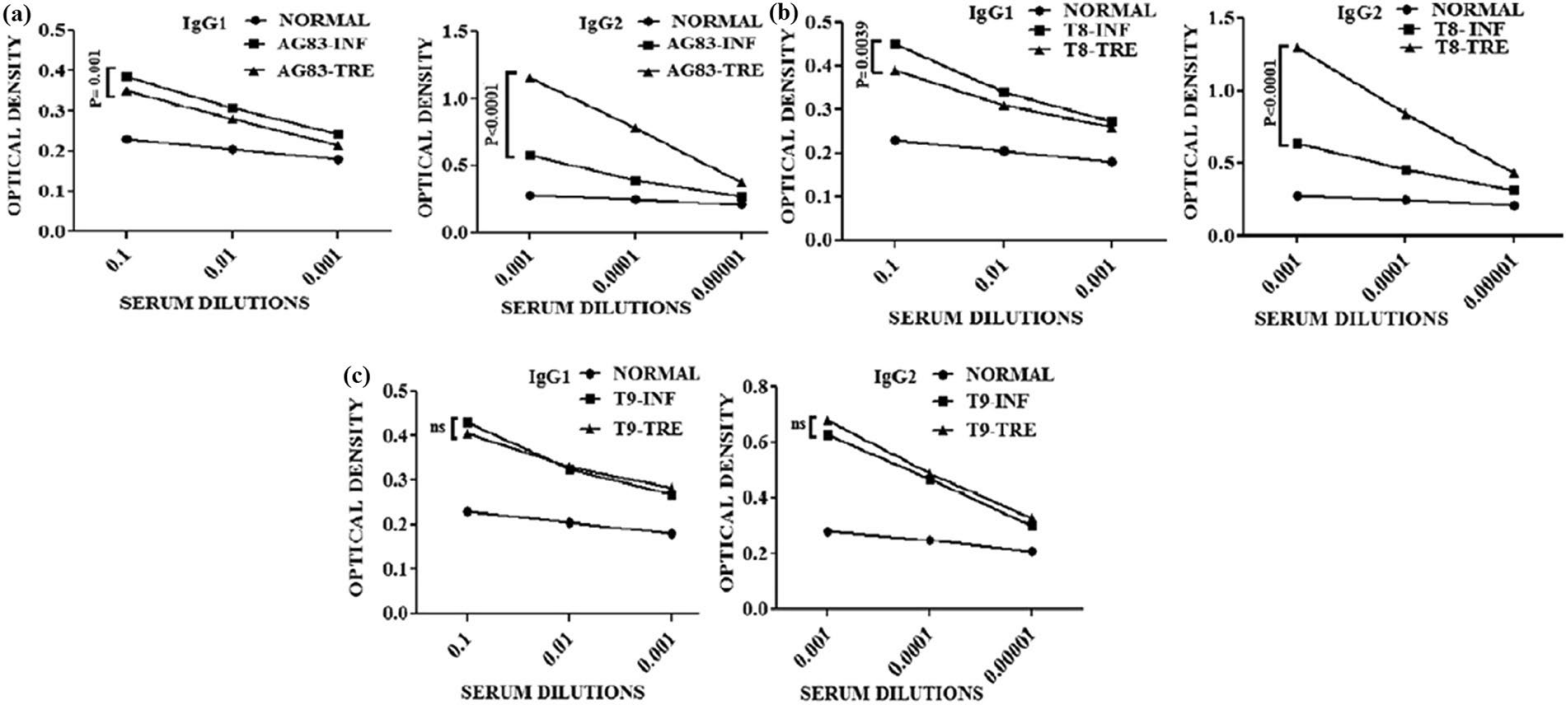
Measurement of antileishmanial IgG1 and IgG2 antibody titers in different groups of hamsters. (**a**–**c**) The dilutions of sera are 10^−1^, 10^−2^, 10^−3^ fold were used to analyze IgG1 of infected and MIL treated groups of hamster. (**a**–**c**) The dilutions of sera are 10^−3^, 10^−4^, 10^−5^ fold were used to analyze IgG2 of infected and MIL treated groups of hamster. Unpaired two-tailed Student’s t-test was performed and levels of significance are indicated by P values; ns. Non significant.

To understand the disease dynamics, anti leishmanial antibodies were measured using the sera from all the groups of animals. The presence of anti leishmanial IgG2 antibodies in the sera of these animals were detected together with IgG1 (Fig. 6a–c). Following MIL treatment, in respect to the infected groups of hamsters (AG83-INF and T8-INF groups), the level of IgG1 isotype of treated groups [AG83-TRE (P = 0.001 at 10^−1^dilution), T8-TRE (P = 0.0039 at 10^−1^dilution)] were decreased significantly (Fig. 6a,b) except for T9-TRE group (hamsters infected with putative MIL-R isolate T9 and then treated with MIL) (Fig. 6c). On the other hand, MIL treated hamsters groups: AG83-TRE (P < 0.0001 at 10^−3^dilution) and T8-TRE (P < 0.0001 at 10^−3^ dilution) showed significantly increased level of IgG2 in respect of AG83-INF and T8-INF groups (Fig. 6a,b). In contrast, there was no significant change in the level of IgG2 between T9-TRE and T9-INF group (Fig. 6c).

### Analysis of Th1/Th2 mRNA cytokines levels by Real-time PCR

**Figure 7:**
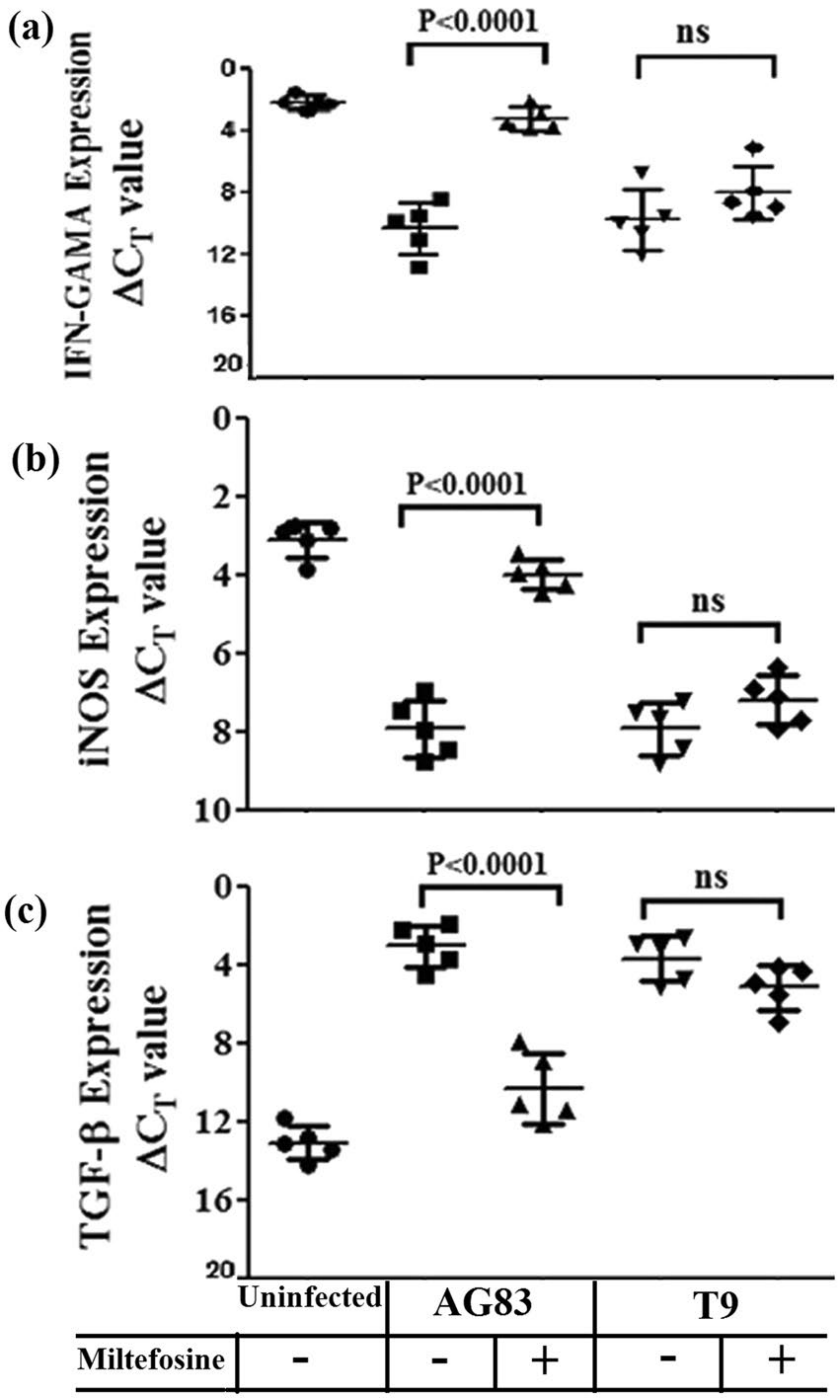
Real-time PCR analysis of mRNAs of Th1 and Th2 cytokines. (**a**) IFN-γ (**b**) iNOS **(c)** TGF-β transcripts were analyzed from uninfected, infected and MIL treated groups of hamsters. Data represents mean ± SD of 5animals per group; unpaired two-tailed Student’s t-test was performed and levels of significance are indicated by P values; ns. Non significant.

Real-time PCR data revealed that there was noteworthy increased expression levels in the mRNA transcripts of IFN-γ (Fig. 7a, panel AG83), iNOS (Fig. 7b, panel AG83) and significant decreased expression levels in the mRNA transcripts of TGF-β (Fig. 7c, panel AG83) in the MIL treated MIL-S group of animals (AG83-TRE). T9-TRE group of animals showed no significant change in the expression levels of mRNA transcripts of Th1, Th2 cytokines and iNOS with respect to T9-INF group (Fig. 7a–c, panel T9).

## Discussion

The common pentavalent antimonials like sodium stibo gluconate (SSG) used for the treatment of VL, necessitate prolonged course of treatment and is losing its efficacy due to increasing parasite resistance. This has emerged as a major difficulty in the treatment and control of VL^9^. The progression of SSG resistance in the endemic region specially in Bihar, directed that resistance could also come out to the other established anti leishmanial drugs and this may be due to poverty, illiteracy, poor health and HIV/VL-coinfection^6, 40, 41^.

Miltefosine had been approved in India in 2002 for the treatment of Indian VL patients^18^, especially cases unresponsive to antimonials and within few years, unresponsiveness to MIL in VL patients^20, 21^ and failure of MIL in the treatment of KA in Nepal have been reported^22^. Recently, two MIL-R *L. donovani* isolates collected from confirmed VL patients of India, were identified by *in vitro* and genome studies^26^. Studies related to patients unresponsive towards MIL, laboratory confirmed MIL-R *L. donovani* and experimentally induced MIL-R *L. donovani* have been executed previously to demonstrate the role of probable factors in the treatment failure and mechanism of resistance towards the drug MIL^22, 27^. Function of fatty acid and steroid metabolism in addition to the expression levels of two MIL transporter proteins, LdMT and its specific beta subunit LdRos3^24, 25^ seem responsible for the resistance. It was further suggested that LdMT gene mutation could be employed as a molecular marker for MIL-R *L. donovani* isolates^26^.

Earlier studies confirmed that the biochemical actions for SSG uptake are catalyzed either by thiol metabolising genes or antimony transporter genes and several types of ATP Binding Cassette (ABC) transporters are related to multi-drug resistance (MDR)^36^. We have noticed the increment of 3.4 times of MRPA gene in the putative MIL-R corroborating the observations that in *in vitro* conditions, the MDR-related proteins [Multidrug Resistant Protein A (MRPA) or P-glycoprotein (PGPA)] have been amplified in different *Leishmania spp*. in response to different drugs^42^. The parasites are not able to metabolize MIL itself and can extrude via either exocytosis or probably by ABC transporter protein, such as P-glycoprotein (mdr1)^43^. These studies considerably increased our knowledge about Miltefosine resistance in clinical isolates of KA. Further studies need to be performed in the natural populations of *L. donovani* to examine the epidemiology of resistance in order to diminish the harshness of KA. In order to divulge the mechanism of resistance, establishment of *in vivo* animal model is essential. The phenotypic expression in experimental infection model may be extrapolated to that of the KA patients unresponsive to Miltefosine.

By testing the MIL susceptibility of all the clinical isolates of KA and PKDL *in vitro* amastigote-macrophage model, we identified one putative MIL resistant (MIL-R) field isolate (study code T9), which is typified as *L. donovani* by ITS1 RFLP and hsp70 RFLP methods. This finding was supported through the measurement of Th1, Th2 cytokines and level of ROS and NO release from MIL induced macrophages infected with this isolate and then treated with the drug. The measurement of ΔΨm and FITC-conjugated Annexin-V and PI double staining results further corroborated our claim. Experimental MIL-resistant *L. donovani* isolates showed down regulated expression of LdMT and LdRos3 transporters^25^. Our study with single field isolate, resistant to MIL also showed the down regulation in the expression of these transporters. The MIL-R fields isolate also showed approximately 3.4 times higher MRPA expression level than MIL-S isolate (Supplementary Fig. S4).

In our attempts to develop the animal model for MIL-R isolate in Golden hamster (*Mesocricetus auratus*), we carried out our *in vivo* study with three clinical isolates: AG83 (MIL-S & SSG-S); T8 (MIL-S & SSG-R) and T9 (MIL-R & SSG-S). Hamster is a superior model for VL and expands a progressive, fatal disease which is very closely related to the human symptoms of the disease^44, 45^. The splenic and hepatic stamps smears revealed the retention of parasite load after MIL treatment in the AG83-TRE and T8-TRE groups of animals of about 3–8%. On contrary, animals infected with MIL-R isolate and then treated with the drug showed retention of 36–69% (average 51.35%) parasites in spleen and 32–62% (average 51.54%) parasites in the liver.

Cell mediated immunity impaired in *Leishmania* infection is characterized by marked T-cell anergy specific for *Leishmanial* antigen^46^. After checking the effects of MIL on treated animals, we became interested to see whether the T-cell anergy occurred during progressive infection, could be reversed by the treatment of MIL. The splenocytes of T9-TRE group of animals failed to proliferate anti leishmanial T-cell in response to leishmanial antigen.

Active VL is also associated with the production of an altered level of antibody^44^. Significant increase in IgG2 levels in cured animals is surrogate marker of enhanced cell mediated immunity^45^. Our study revealed that MIL treated hamsters groups: AG83-TRE and T8-TRE showed significantly increased level of IgG2, indirectly indicating development of an effective Th1 type immune response^44, 47^. In contrast, there was no enhancement of cell mediated immunity in the treated MIL-R infected (T9-TRE) group. This observation further supported through the measurement of mRNA transcripts of Th1 and Th2 cytokines.

Our observations strongly suggested that out of three groups of experimental animals, AG83-TRE and T8-TRE groups of animal were almost cured with MIL treatment but T9-TRE group of animals did not realize recovery establishing the animal model for MIL-R faithfully.

The whole genome analysis of this clinical isolate is in progress.

## Methods

### Ethics Statements

Bone marrow aspirates were collected from KA patients and approved by the Ethical Committee of the Calcutta National Medical College, Kolkata. The written consent was obtained from legal guardian of the patient (as it was the case of a minor). In the present study, all methods were carried out in accordance with the relevant guidelines.

### Clinical isolate from KA Patient and Reference strains

Information concerning the patients’ detail (n = 26) were reported in our previous studies^4, 5, 34, 36, 48^ and rest (n = 1) was mentioned in supplementary Table SI. *L. donovani* isolate, AG83 (MHOM/IN/83/AG83) was used as MIL and SSG sensitive standard^35, 36^. Presence of amastigotes in the bone marrow of the patients were established by Giemsa staining as well as transforming them into promastigote forms in culture medium M199 supplemented with 10% Fetal Bovine Serum (FBS).

### Characterization of the isolates by PCR-RFLP method

Genomic DNA was prepared from samples and then categorized them by ITS1-RFLP and hsp70-RFLP methods by using 5UHaeIII (Bioenzyme, USA) enzyme^5, 32, 34, 49^.

### Cell line used for *in vitro* study

Murine Macrophage (MØ) like tumor cell, RAW 264.7 was maintained in complete RPMI 1640 medium with 10% FBS at 37 °C with 5% CO_2_ in a humidified atmosphere.

### *In vitro* drug sensitivity assay and determination of EC_50_ value

Drug sensitivity of intracellular amastigotes was evaluated as stated earlier^50^. RAW 264.7 cells (MØs) infected with *Leishmania* isolates were treated either with various concentrations of MIL [0.1 μM, 0.3 μM, 1 μM, 3 μM, 10 μM and 30 μM] or SSG [0.01 μg/mL (0.013 μM), 0.1 μg/mL (0.134 μM), 1 μg/mL (1.34 μM), 10 μg/mL (13.41 μM), 100 μg/mL (134.1 μM)]^34^ and untreated MØs received only medium. At the endpoints, experimental cover slips were washed and stained with 10% Giemsa (Sigma) and examined under microscope. One hundred MØs per cover slip were scored and no. of intracellular parasites was determined. The values of half maximal effective concentration (EC_50_) for each of the isolates were anticipated against the both of drugs using GraphPad Prism Software (San Diego, California, USA).

### Assay of cytokines

Drug treatments were given to the MØs infected with putative MIL-R isolate T9 along with MIL-S isolates T8 (KA isolate which is SSG-R)^34^ and AG83 at different concentrations of MIL [0.3 μM, 3 μM & 30 μM]. Then supernatants were collected to measure Th1 (IL-12 and TNF-α) and Th2 (IL-10) cytokines using ELISA kit (BD Biosciences, CA, USA) according to the manufacturer’s protocol.

### Quantification of nitric oxide (NO)

NO production was evaluated by the Griess Reagent as described previously^39^.

### Measurement of reactive oxygen species (ROS)

Reactive Oxygen Species (ROS) measured by the cell permeable, nonpolar, H_2_O_2_ sensitive probe 2′,7′- Dichlorofluorescein Diacetate (H_2_DCFDA) as previously described^39^.

### Measurement of mitochondrial trans membrane potential

Transmembrane potential (ΔΨm) was evaluated using JC-1, a lipophilic cationic dye^39^. Untreated and MIL treated (treated with MIL at a concentration of 40 μM for 72 hrs) *Leishmania* promastigotes were incubated with JC-1 for 25 min in dark and then washed. Cell pellets were resuspended in PBS and subjected to Flow Cytometry analysis in a BDFACSAria II cell sorter using an excitation wavelength of 488 nm and emissions at 530 nm for green and 590 nm for red fluorescence after appropriate fluorescence compensation and analyzed by FACSDIVA software (BD Biosciences, San Jose, CA, USA).

### Annexin V fluorescein isothiocyanate (FITC)- Propidium Iodide (PI) Assay

Double staining for FITC Annexin V*-*PI was performed as demonstrated earlier^39^. Briefly, untreated, MIL-treated (72 hrs treatment) promastigotes were washed with PBS. The pellets were resuspended in 1X binding buffer at a concentration of 1 × 10^6^/ml followed by incubation with 5 μl of FITC Annexin V and 1 µg/ml PI for 25 min in dark. Then 400 μl of 1X binding buffer was added and samples were analyzed by Flow Cytometry within 1 hr.

### RNA isolation and semi quantitative RT-PCR analysis of the genes responsible for MIL transport

RNA was isolated from samples by disrupting in Trizol solution^51^ and newly prepared cDNA were then amplified by taking 0.5 μl of cDNA with1 μl 10 mM dNTP, 1.5 μl 50 mM MgCl_2_, 0.5 μl Taq polymerase as well as gene specific primers (Supplementary Table S2). Amplification reactions were performed with cycling conditions for genes of interest were 5 min at 95 °C, followed by 30 cycles of denaturation at 95 °C for 30 s, annealing at (55°–60 °C) for 30 s and extension at 72 °C for 30 s. PCR-amplified respective gene products were checked by Agarose gel electrophoresis. For Densitometry analyses, the ImageJ software (National Institute of Health) was used and the same band area in Agarose gel was used to find out band intensity and normalized for GAPDH.

### Statistical analyses for the *in vitro* study

Results of all *in vitro* studies are expressed as mean ± SD. Student’s t test for significance was carried out using GraphPad prism software and P value of <0.05 was considered to be significant. Correlation between the EC_50_ and other parameters were determined by Spearman rank correlation coefficient and expressed as r^36^.

### Animals for *in vivo* study

Golden hamsters (*Mesocricetus auratus*) reared in Institute facilities, were used for the present study with prior approval of the Animal Ethics Committee of the Indian Institute of Chemical Biology, Kolkata, India. All animal experiments were carried out according to the National Regulatory Guidelines issued by Committee for the Purpose of Control and Supervision of Experiments on Animals (CPCSEA), Ministry of Environment and Forest, Govt. of India.

### *Leishmania* isolates used for *in vivo* study

For the present study, we have taken putative MIL-R (T9) along with T8 and AG83. The isolates were used for comparative analysis.

### Selection of MIL doses for *in vivo* experiments

Hamsters were challenged with freshly transformed *Leishmania* T9, T8 and AG83 parasites (10^7^parasites/animal) via intra cardiac route^52^. After 8 weeks of infection, infected animals were treated with MIL at dose of 40 mg/kg (body weight) for 10 consecutive days and were sacrificed on days 45 post treatment^44^. Hamsters have been grouped in the following ways: Normal: 5 healthy control without challenge infection and treatment with MIL; Infected: each 10 animals were infected with T9, T8 and AG83 respectively and would be represented as T9-INF, T8-INF and AG83-INF respectively; Treated: among 10 infected animals of each group, 5 were treated with MIL. These groups henceforth would be written as T9 -TRE, T8-TRE and AG83-TRE respectively.

### Collection of blood and preparation of serum

Blood was collected from hamsters as illustrated earlier^39^ and kept overnight at 4 °C. Then serum was prepared from collected blood sample.

### Determination of Organomegaly and Parasite Burden in spleen and liver

Weight and parasitic burden of spleen and liver from experimental groups of animals were assessed after sacrifice. Splenic and hepatic parasite burden of hamsters of different groups were determined by microscopic evaluation of Giemsa stained tissue imprints method^39^. Total parasite load in each organ is expressed in LDU unit (Leishman-Donovan Unit). 1 LDU = amastigote per nucleated cell x organ weight in milligram.

### Preparation of soluble Leishmanial antigen (SLA)

Leishmanial lysates from washed promastigotes (10^9^/ml) were prepared by several cycles (minimum six) of freezing (−70 °C) and thawing (37 °C) followed by five minutes of incubation on ice^53^.

### T-cell proliferation assay

Splenocytes from studied groups of hamsters were prepared after Ficoll density gradient centrifugation and dissolved in complete RPMI medium and then plated in 96-well plates (at a concentration of 10^5^ cells/well) therefore allowed to proliferate for 72hrs at 37 °C with 5% CO_2_ either in the presence or absence of SLA (5 μg/ml) or ConA (5 μg/ml). Cells were treated with MTT (0.5 mg/ml) as described earlier^39^. Then Isopropanol-HCl mixture (0.04%) was used to solubilise the MTT crystals and the absorbance at 570 nm was interpreted at an ELISA plate reader (DTX 800 multimode detector, Beckman Coulter, California).

### Measurement of anti leishmanial antibody responses

Serum samples were collected from different groups of hamsters and examined to find out the parasite SLA-specific antibody titer. IgG1 and IgG2 present in the collected sera were measured as stated earlier^39^.

### Real-time PCR to estimate expressions mRNAs of Th1/Th2 cytokines levels

RNA was isolated from the splenocyte of hamsters and 50–100 ng of total RNA was used for synthesis of cDNA. RT-qPCR was done as described elsewhere^44^. Briefly, it was carried out with 7 μl of SYBR green PCR master mix, 1 μl of cDNA from RT reaction mix and gene specific primers in a final volume of 15 μl. PCR was conducted under the following conditions: initial denaturation at 95 °C for 10 min followed by 40 cycles, each consisting of denaturation at 95 °C for 15 s, annealing at 58 °C for 1 min and extension at 72 °C for 40 s per cycle using the ABI 7500 Real time PCR system and data were analyzed by the comparative CT method^45, 54^. cDNAs from infected hamsters were used as comparator samples. All quantifications were normalized to the housekeeping gene hypoxanthine phosphoribosyl transferase (HPRT).

### Statistical analysis for *in vivo* study

Statistical level of significance between different groups was calculated by unpaired two-tailed Student’s t-test with GraphPad Prism software (San Diego, California, USA). P < 0.05 were considered to be significant for all analyses.

## Electronic supplementary material

Supplementary info
