# Supplementary material for: Miltefosine Resistant Field Isolate From Indian Kala-Azar Patient Shows Similar Phenotype in Experimental Infection
Source: Sci Rep. 2017 Sep 4;7:10330. doi: 10.1038/s41598-017-09720-1 (PMC5583325; doi:10.1038/s41598-017-09720-1)
Supplement: Supplementary file 1 — Supplementary info [file 41598_2017_9720_MOESM1_ESM.pdf]

Miltefosine Resistant Field Isolate From Indian Kala-Azar Patient Shows Similar Phenotype in Experimental Infection

Supriya Khanra[1,a], Nibedeeta R. Sarraf[1], Anjan K. Das[2], Syamal Roy[3,b,*] & Madhumita Manna[1,c,*]

[1] Department of Zoology, Barasat Govt. College, 10, K.N.C Road, Kolkata 700124, India

[2] Department of Pathology, Calcutta National Medical College, 32, Gorachand Road, Kolkata 700014, India

[3] Department of Infectious Diseases & Immunology, Indian Institute of Chemical Biology, 4, Raja S.C. Mullick Road, Kolkata 700032, India

[a] Current address: Crystallography and Molecular Biology Division, Saha Institute of Nuclear Physics, 1/AF Bidhannagar, Kolkata 700064, India

[b] Current address: Cooch Behar Panchanan Barma University, Vivekananda Road, Cooch Behar, West Bengal 736101, India

[c] Current address: Bidhannagar College, EB 2, Salt Lake, Sector I, Kolkata 700064, India

*Corresponding authors

Dr. Madhumita Manna, Principal (WBSES), Bidhannagar College, EB 2, Sector 1, Salt Lake, Kolkata 700064, India, Phone No. +919903072249

Email: madhumita.manna09@gmail.com

Prof. Syamal Roy, Vice Chancellor, Cooch Behar Panchanan Barma University, Vivekananda Road, Cooch Behar, West Bengal 736101, India, Phone No. +919874532967

Email: drsyamalroy@yahoo.com

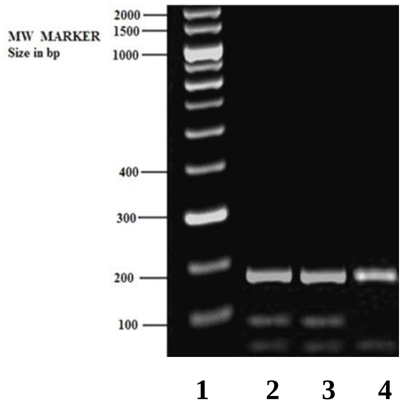

Supplemental data Figure 1. The amplified ITS1 region of nuclear DNA of the clinical isolate of KA (T9) along with DD8 and K27 were digested using restriction enzyme Hae III. Lane 1, MW marker (100 bp); lane 2, DD8; lane 3, T9; lane 4, K27.

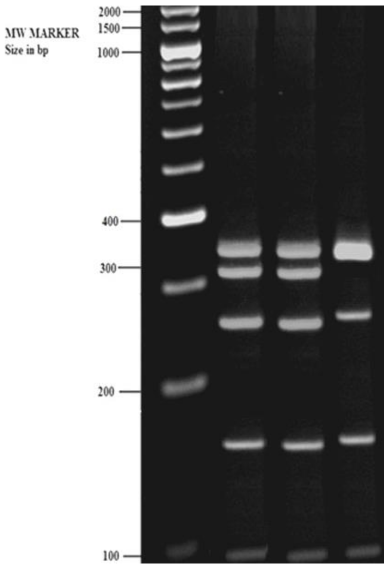

Supplemental data Figure 2. The amplified hsp70 region of nuclear DNA of the clinical isolate of KA (T9) along with DD8 and K27 were digested using restriction enzyme Hae III. Lane 1, MW marker (100 bp); lane 2, DD8; lane 3, T9; lane 4, K27.

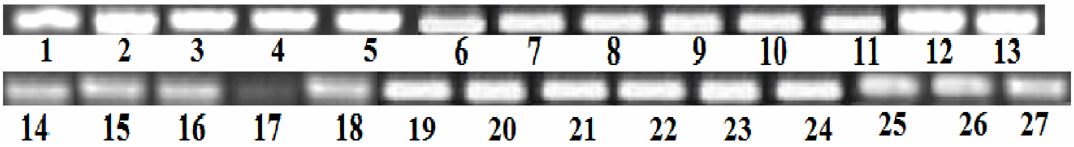

(a) LdMT mRNA expressions

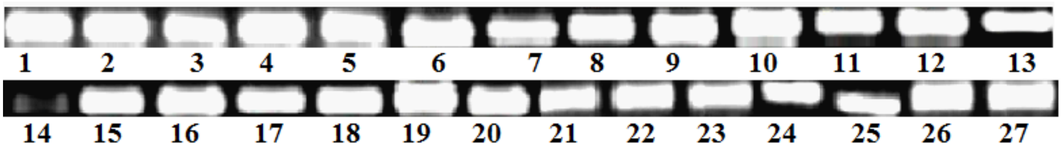

(b) LdRos3 mRNA expressions

Supplemental data Figure 3. (a) Gel images of LdMT mRNA expressions. Lanes; 1, AG83; 2, T2; 3, T3; 4, T7; 5, M1; 6, BHU 573; 7, BI; 8, BHU 581; 9, BHU 568; 10, BHU 575; 11, P2; 12, RAJ-04; 13, BHU 569; 14, T4; 15, BHU 574; 16, BHU 572; 17, T9; 18, RAJ-07; 19, PG4; 20, RAJ-05; 21, P1; 22, BHU 592; 23, BHU 965; 24, T8; 25, PG2; 26, PG3; 27, BHU 860. (b) Gel images of LdRos3 mRNA expressions. Lanes; 1, AG83; 2, M1; 3, RAJ-05; 4, T8; 5, P1; 6, T2; 7, T4; 8, BHU 592; 9, PG4; 10, T3; 11, PG2; 12, BHU 965; 13, PG3; 14, T9; 15, BHU 572; 16, BHU 569; 17, BHU 860; 18, RAJ-04; 19, T7; 20, BHU 568; 21, BHU 573; 22, BHU 575; 23, P2; 24, BHU 574; 25, BHU 581; 26, BI; 27, RAJ-07.

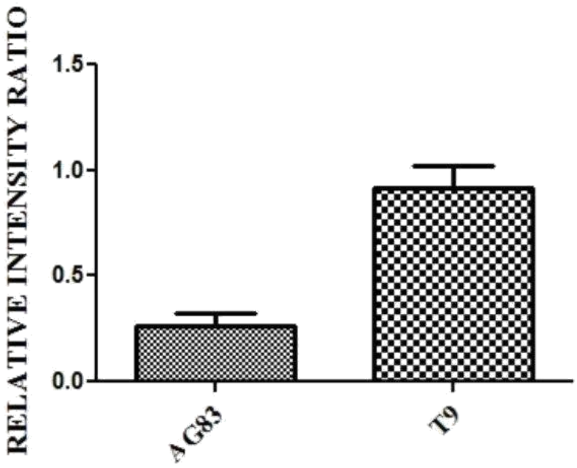

Supplemental data Figure 4. Graphical representation of the densitometric data. Expression level of MRPA was expressed as a ratio of MRPA mRNA level to GAPDH mRNA level. Data was expressed as the mean ± SD of three independent experiments.

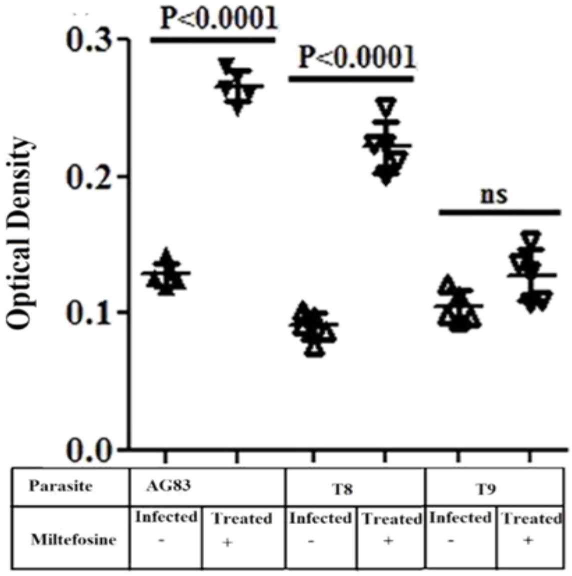

Supplemental data Figure 5. Expansion of antileishmanial T cells. Splenocytes isolated from different groups of infected and MIL drug treated animals were stimulated with SLA and the resulting proliferation of splenocytes were assayed using MTT cell viability assay. Data represents mean ± SD of 5animals per group; unpaired two-tailed Student’s t-test was performed and levels of significance are indicated by P values; ns. Non significant.

Description	Details of the patient
**Study code[a]**	T9
**Age[b]**	5 Years
**Sex[c]**	Female
**Country[d]**	India
**Clinical History[e]**	Anemia, fever for 3 months
**Spleen size[f]**	7cm
**Liver size[g]**	3 cm
**Bone marrow parasite status[h]**	Positive (as shown in the picture, provided below the Supplemental data Table 1)
**TC[i]**	3,200/C .m.m
**Haemoglobin levels[j]**	7.2gm%
**Neutrophils[k]**	52%
**Lymphocytes[l]**	34%
**Monocyte[m]**	08%
**Eosinophils[n]**	06%
**Basophils[o]**	0.0%
**ESR[p]**	20mm
**Chest X-ray[q]**	Within normal limit
**LFT & RFT[r]**	Within normal limit
**Diagnosis[s]**	Kala-azar
**Treatment administered[t]**	Amphotericin B

Supplemental data Table 1. Clinical details of the KA patient used for the present study

[a]Study code given to the clinical isolate of KA for the present study from whom the Mil-Resistant strain was isolated.

[b]Age of the KA patient

[c]Sex of the KA patient

[d]Country of the patient

[e]Clinical history of the patient

[f]Spleen size of the patient

[g]Liver size of the patient

[h]Bone marrow parasite status of the patient

[i]TC count of the patient

[j]Haemoglobin levels of the patient

[k]Neutrophils count of the patient

[l]Lymphocytes count of the patient

[m]Monocyte count of the patient

[n]Eosinophils count of the patient

[o]Basophils count of the patient

[p]ESR of the patient

[q]Chest X-ray of the patient

[r]Liver Function Test (LFT) & Renal Function Test (RFT) of the patient

[s]Diagnosis of the patient
[t]Treatment administered to the patient

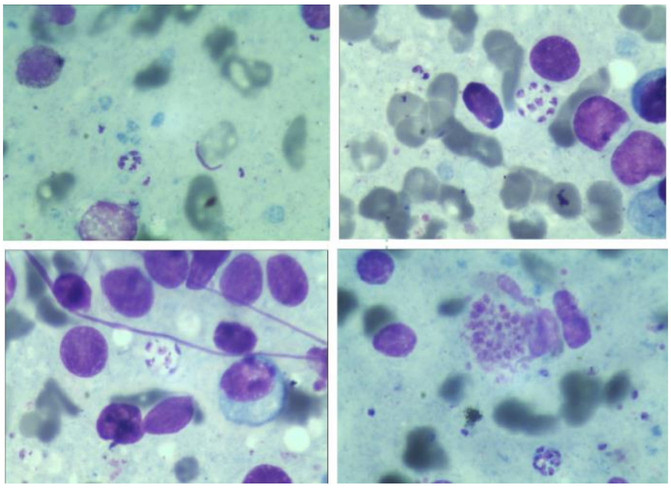

Images of Giemsa-stained amastigotes; present in bone marrow aspirate of the patient from whom the clinical isolate (T9) was collected

**Studied gene**	**Primer sequence (5’-3’)**
MIL transporter LdMT	F-CAAGTGCCTTTCCACCAGAATC<br>R-CTCACCTTTTTGAACTCCAACAGG
LdRos3	F-ACGACACGGCTTGATTTTCG<br>R-GAGTAGTCCACGGAGGCAGTAAAG
Multidrug resistant protein (MRPA)	F-CGAAAGTTGAGCAGGAGAC<br>R-AATCCCCAAGCAGCCAGAC

Supplemental data Table 2. Studied genes and specific primer sequences used in the semi quantitative RT PCR
